# Supplementary material for: Comprehensive analysis of insertion sequences within rRNA genes of CPR bacteria and biochemical characterization of a homing endonuclease encoded by these sequences
Source: J Bacteriol. 2024 Jun 10;206(7):e00074-24. doi: 10.1128/jb.00074-24 (PMC11270868; doi:10.1128/jb.00074-24)
Supplement: Supplemental material — Tables S1-S6 and Fig S1-S7. [file jb.00074-24-s0001.pdf]

Supplementary Materials

Comprehensive analysis of insertion sequences within rRNA genes of CPR bacteria and biochemical characterization of a homing endonuclease encoded by these sequences

Megumi Tsurumaki[a,b], Asako Sato[a], Motofumi Saito[a,b], and Akio Kanai[a,b,c] #

[a] Institute for Advanced Biosciences, Keio University, Tsuruoka 997-0017, Japan

[b] Systems Biology Program, Graduate School of Media and Governance, Keio University, Fujisawa 252-0882, Japan

[c] Faculty of Environment and Information Studies, Keio University, Fujisawa 252-0882, Japan

#Corresponding Author

Akio Kanai, PhD

Institute for Advanced Biosciences, Keio University

Tsuruoka, Yamagata 997-0017, Japan

Tel: +81-235-29-0524

Fax: +81-235-29-0525

E-mail: akio@sfc.keio.ac.jp

Running title: Intron-encoded homing endonucleases in CPR bacteria

Keywords: Candidate Phyla Radiation, rRNA intron, homing endonuclease, bioinformatics

Table S1. Summary of the number of CPR bacterial genomes which encoded rRNA gene sequences used in this study.

Taxonomy	Phyla	Complete genomes	Draft genomes
Microgenomates Group (MG)	14	9	161
Parcubacteria Group 1 (PB1)	9	2	85
Parcubacteria Group 2 (PB2)	5	0	42
Parcubacteria Group 3 (PB3)	5	1	38
Parcubacteria Group 4 (PB4)	9	6	114
Others in Parcubacteria Group (Other PB)	9	5	92
Ca. Katanobacteria (WWE3)	1	1	15
Ca. Saccharibacteria (Sa)	1	34	9
Ca. Peregrinibacteria (Pe)	1	8	20
Others	11	6	37
**Total**	**65**	**72**	**613**

Table S2. The number of rRNA genes with and without ISs

Taxonomy	Total number of 16S rRNA genes	The number of 16S rRNA genes with ≥100bp ISs	Total number of 23S rRNA genes	The number of 23S rRNA genes with ≥100bp ISs
Microgenomates Group (MG)	97	54 (56 %)	83	70 (84 %)
Parcubacteria Group 1 (PB1)	64	22 (34 %)	50	33 (66 %)
Parcubacteria Group 2 (PB2)	19	13 (68 %)	20	17 (85 %)
Parcubacteria Group 3 (PB3)	26	11 (42 %)	22	18 (82 %)
Parcubacteria Group 4 (PB4)	40	29 (73 %)	35	34 (97 %)
Others in Parcubacteria Group (Other PB)	37	26 (70 %)	41	38 (93 %)
Ca. Katanobacteria (WWE3)	11	2 (18 %)	11	6 (55 %)
Ca. Saccharibacteria (Sa)	37	15 (41 %)	35	35(100%)
Ca. Peregrinibacteria (Pe)	23	0 (0 %)	22	14 (64 %)
Others	26	11 (42 %)	29	19 (66 %)
Total	380	183 (48 %)	348	284 (82 %)

Table S3. Summary of the classification of the ISs in CPR bacterial rRNA genes

	ISs in 16S rRNA genes			ISs in 23S rRNA genes		
	group I intron	group II intron*	No hits to group I or II intron	group I intron	group II intron	No hits to group I or II intron
**Encoding ORFs**	**85**	**39**	**52**	**211**	**0**	**66**
23S_rRNA_IVP	3	0	15	2	0	2
GIY-YIG	0	0	15	0	0	15
LAGLIDADG_1	4	39	8	165	0	31
LAGLIDADG_2	43	0	10	44	0	18
LAGLIDADG_3	35	0	4	0	0	0
**No annotated ORFs**	**49**	**14**	**139**	**222**	**0**	**469**
**Total**	**134**	**53**	**191**	**433**	**0**	**535**

*** Including partial hits**

Table S4. Summary of the classification of the ORFs encoded in the rRNA ISs of CPR bacteria

Protein domains (Pfam ID)	ORFs in 16S rRNA gene ISs	ORFs in 23S rRNA gene ISs	Total
23S_rRNA_IVP (PF05635)	18	4	**22**
GIY-YIG (PF01541)	15	15	**30**
LAGLIDADG_1 (PF00961)	51	197	**248**
LAGLIDADG_2 (PF03161)	54	62	**116**
LAGLIDADG_3 (PF14528)	39	-	**39**
**Total**	**177**	**278**	**455**

Table S5. List of query sequences for LAGLIDADG proteins

Protein name	Length	Protein name
I-AabMI	244	*Ascocalyx abietina*
I-AniI	254	*Aspergillus nidulans*
I-CeuI	218	*Chlamydomonas eugametos*
I-CkaMI	306	*Cordyceps kanzashiana*
I-CpaMI	299	*Cryphonectria parasitica*
I-CraMI	302	*Cordyceps ramosopulvinata*
I-CreI	163	*Chlamydomonas reinhardtii*
I-DmoI	194	*Desulfurococcus mobilis*
I-GpeMI	308	*Grosmannia penicillata*
I-GpiI	301	*Grossmania pipceiperda*
I-GzeI	301	*Gibberella zeae*
I-GzeII	298	*Gibberella zeae*
I-HjeMI	254	*Hypcrea jecarina*
I-LtrI	308	*Leptographium truncatum*
I-LtrWI	304	*Leptographium truncatum*
I-MpeMI	309	*Moniliopthora perniciosa*
I-MsoI	170	*Monomastix* sp.
I-OnuI	299	*Ophiostoma novo-ulmi*
I-PanMI	303	*Podospora anserina*
I-SceI	235	*Saccharomyces cerevisiae*
I-SmaMI	302	*Sordaria macrospore*
I-SscMI	300	*Sclerotinia sclerotiorum*
I-Vdi141I	169	*Vulcanisaeta distributa*

The sequences were obtained from the LAGLIDADG Homing Endonuclease Database and Engineering Server (LAHEDES) (http://www.homingendonuclease.net)

Table S6. List of oligonucleotide sequences used in the experiments

Name	Sequence	Comment
*Eco*RI-SP-S	5'-AATTCTGGTGAACGTCAGCAGTAACTCTAACTG TTCTAAG GTAGCGAAGTCCTTTGTCGTGTAA-3'	Oligo with target sequence of *Shapiro* enzyme (sense strand)
*Bam*H1-SP-A	5'-GATCTTACACGACAAAGGACTTCGCTACCTTAG AACAGTT AGAGTTACTGCTGACGTTCACCAG-3'	Oligo with target sequence of *Shapiro* enzyme (antisense strand)
*Eco*RI-KA-S	5'-AATTCGGCGGCCGTAACTATAACGGTCCTAAGG TAGCGAA ATTCCTTGTCGGGTAAGTTCCGAC-3'	Oligo with target sequence of *Kazan* enzyme (sense strand)
*Bam*HI-KA-A	5'-GATCGTCGGAACTTACCCGACAAGGAATTTCGC TACCTTA GGACCGTTATAGTTACGGCCGCCG-3'	Oligo with target sequence of *Kazan* enzyme (antisense strand)

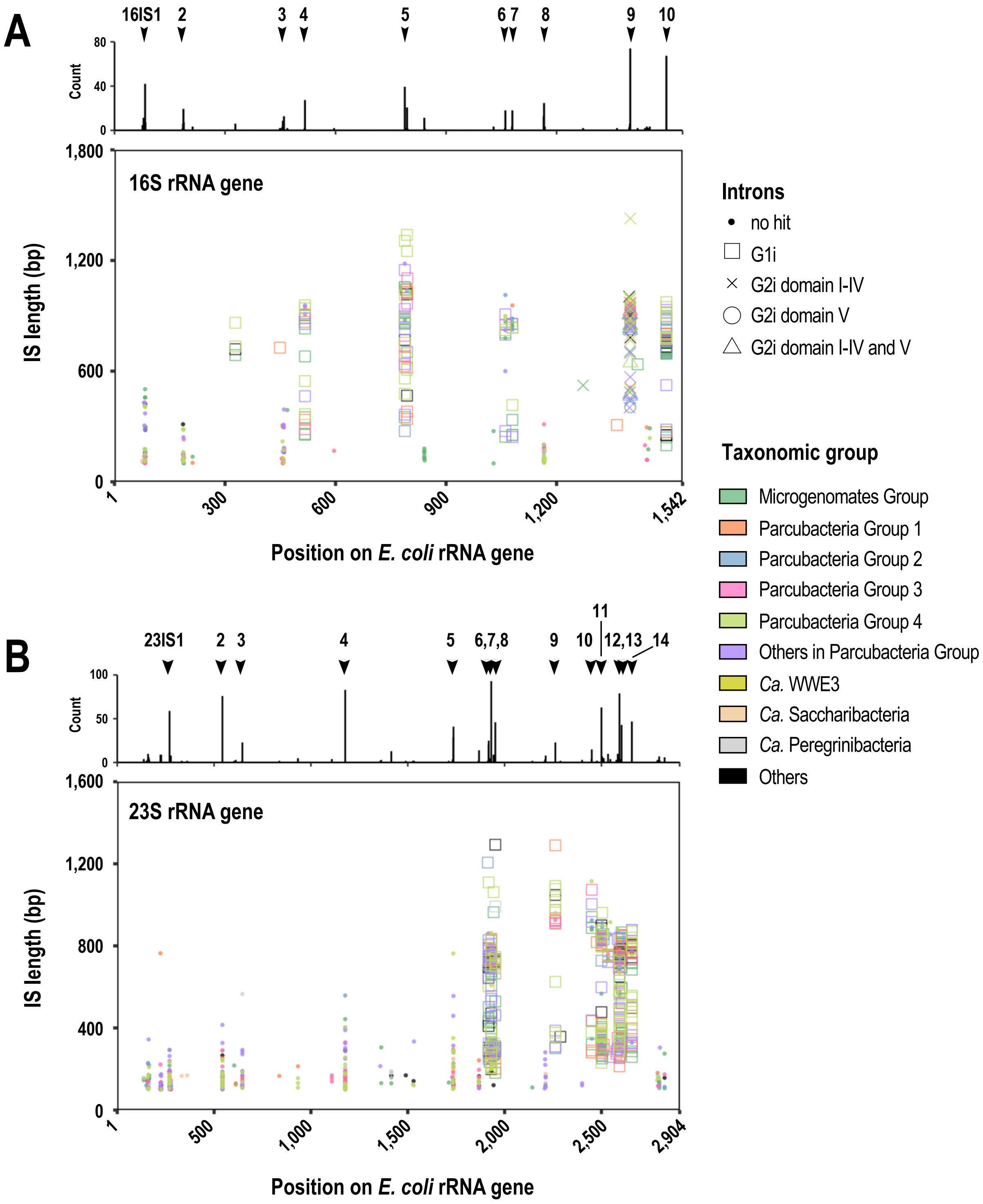

Fig S1. Positions of ISs in rRNA genes of CPR bacteria and the presence of introns. Positions and lengths of inserted sequences in the (A) 16S rRNA and (B) 23S rRNA genes of CPR bacteria are plotted. Plot colors correspond to taxonomic groups, and plot shapes indicate the presence of group I or II introns. Positions at which ISs are frequently found are indicated by black arrows and assigned numbers as follows: 16IS1–10 for 16S rRNA gene, 23IS1–14 for 23S rRNA gene (see also Fig. S3).

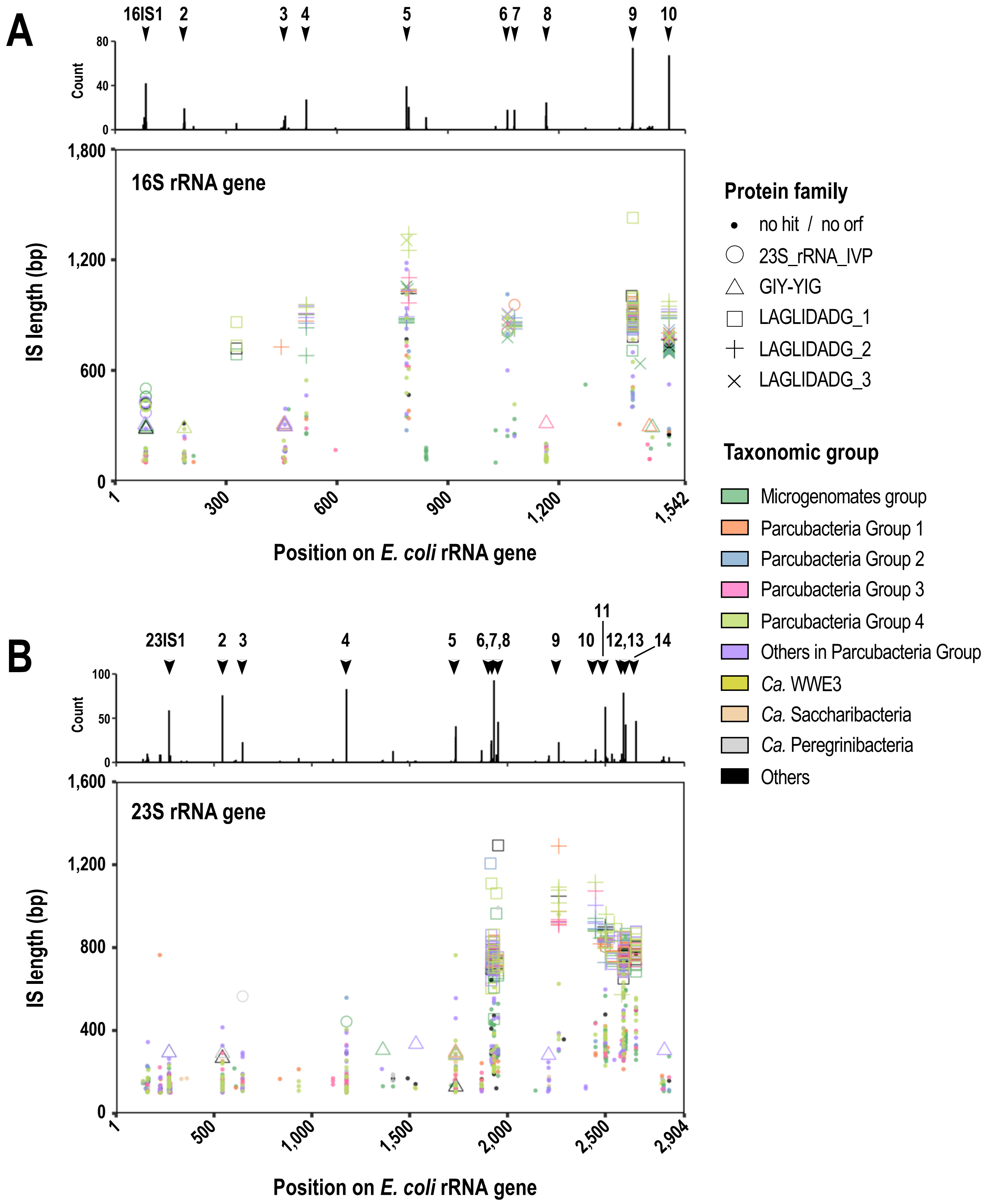

Fig S2. Positions of ISs in rRNA genes of CPR bacteria and classification of the ORFs encoded in the ISs. Positions and lengths of ISs in the (A) 16S rRNA and (B) 23S rRNA genes of CPR bacteria are plotted. Plot colors correspond to taxonomic groups, and plot shapes indicate the Pfam-based annotation of the IS-encoded ORFs. Positions at which the ISs are frequently found are indicated by black arrows and assigned numbers as follows: 16IS1–10 for the 16S rRNA gene, and 23IS1–14 for the 23S rRNA gene (see also Fig. S3).

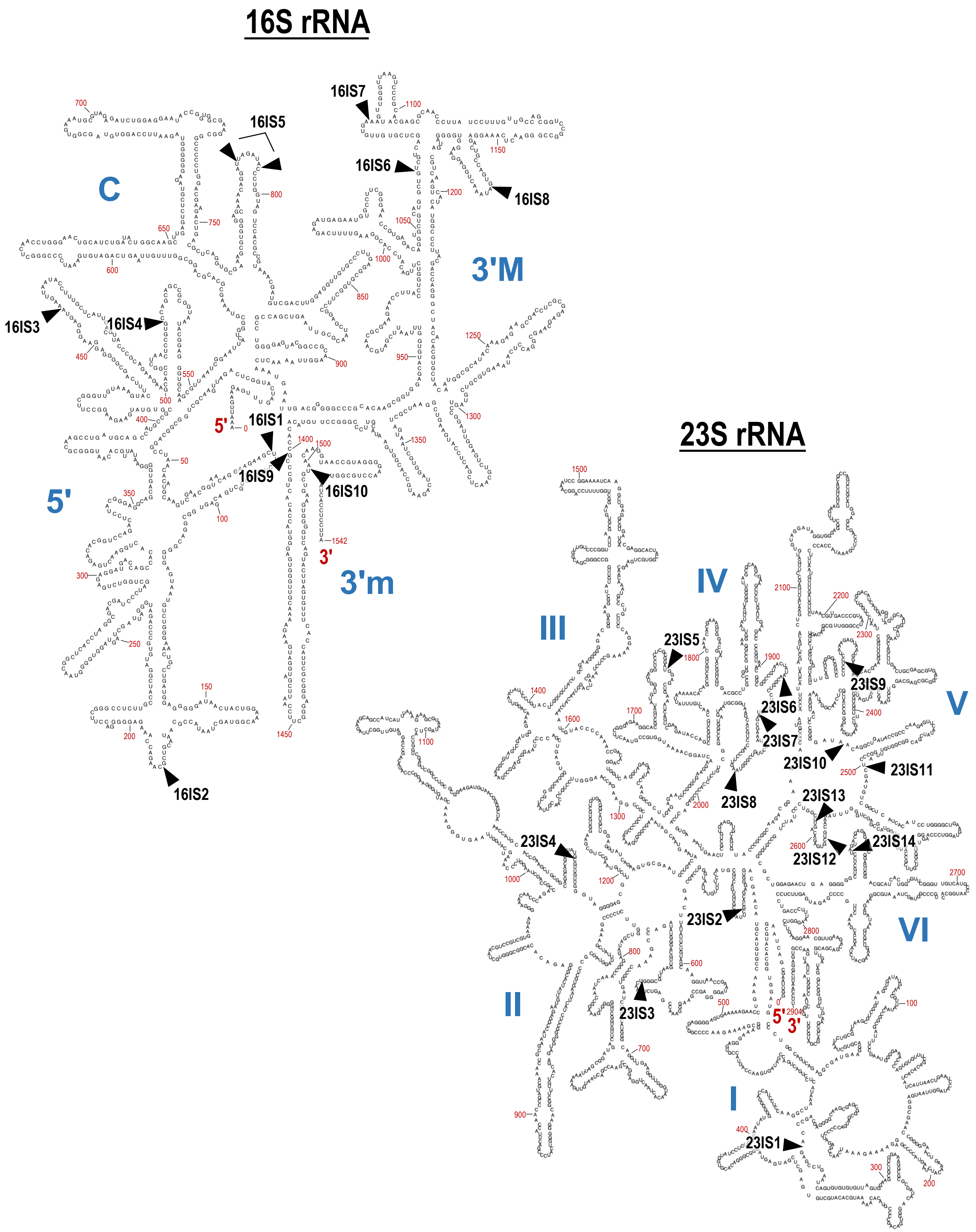

Fig S3. Distribution of insertion sites on each rRNA secondary structure. Locations at which ISs are frequently found (see Fig. S2) are shown on the secondary structures of *E. coli* 16S rRNA and 23S rRNA. Images of rRNA secondary structures were obtained from the Center for Molecular Biology of RNA (http://rna.ucsc.edu/rnacenter/ribosome_images.html).

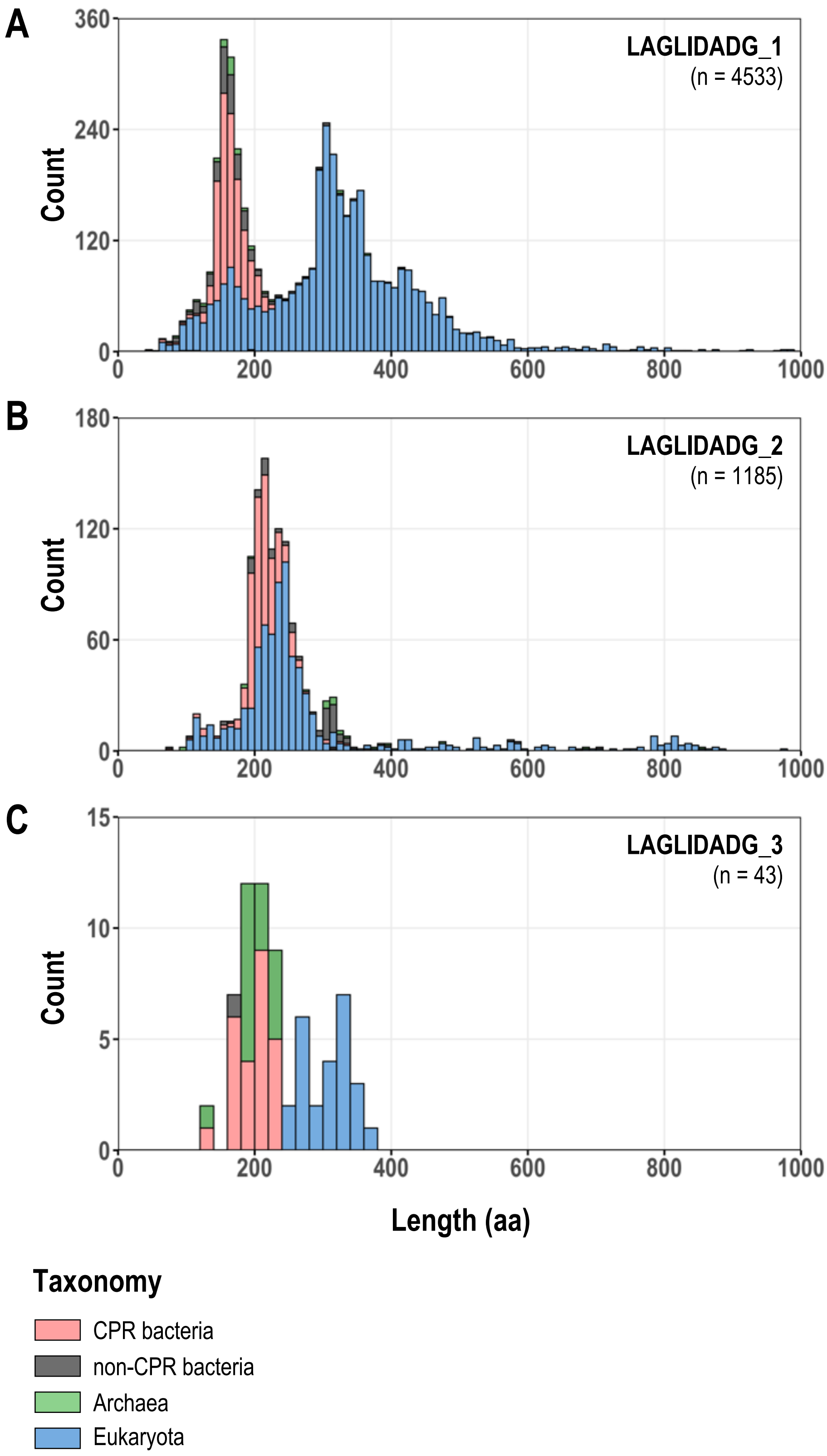

Fig S4. Length distributions of LAGLIDADG proteins for each domain type. Histograms show the size distributions of LAGLIDADG proteins from CPR bacteria and other organisms (see Fig. 2) for each homing endonuclease domain type (A: LAGLIDADG_1; B: LAGLIDADG_2; C: LAGLIDADG_3). Proteins with a combination of different types of LAGLIDADG domains are excluded from the data. Bins are colored according to taxonomic group (CPR bacteria; light red; non-CPR bacteria, dark gray; Archaea, green; Eukaryota, blue). Each horizontal axis is limited to the range in which the main peak occurs.

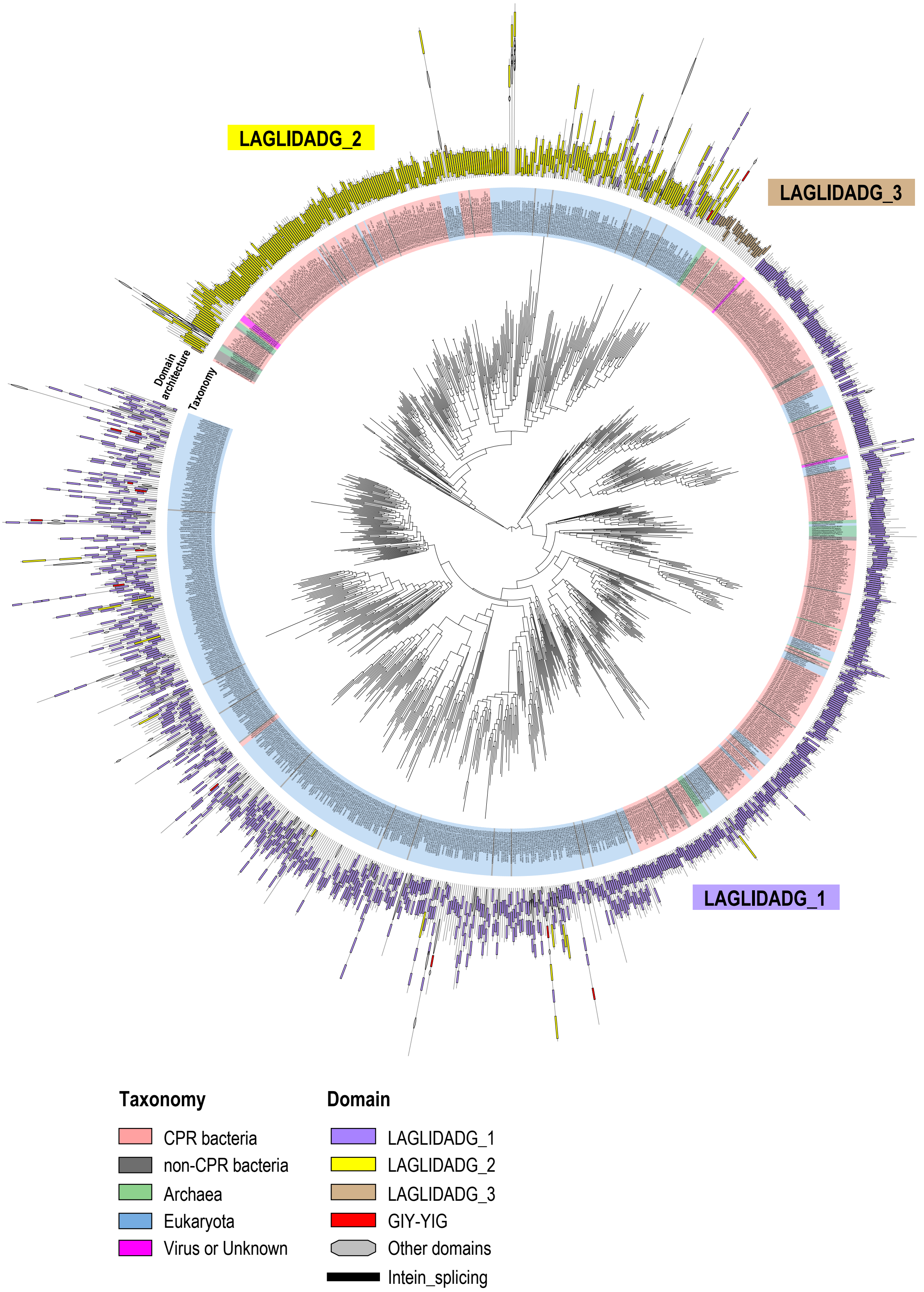

Fig S5. Phylogenetic tree and protein domain architectures of LAGLIDADG proteins in three domains of life. The phylogenetic tree was constructed based on 1074 sequences of LAGLIDADG proteins from different species using the maximum likelihood method and rooted using midpoint rooting. Taxonomic groups of organisms are represented by both colored text and strips: CPR bacteria, light red (n = 427); non-CPR bacteria, dark gray (n = 60); Archaea, green (n = 25); Eukaryota, blue (n = 557); viruses or unclassified, pink (n = 5). Protein domain architectures are shown in the outermost circle.

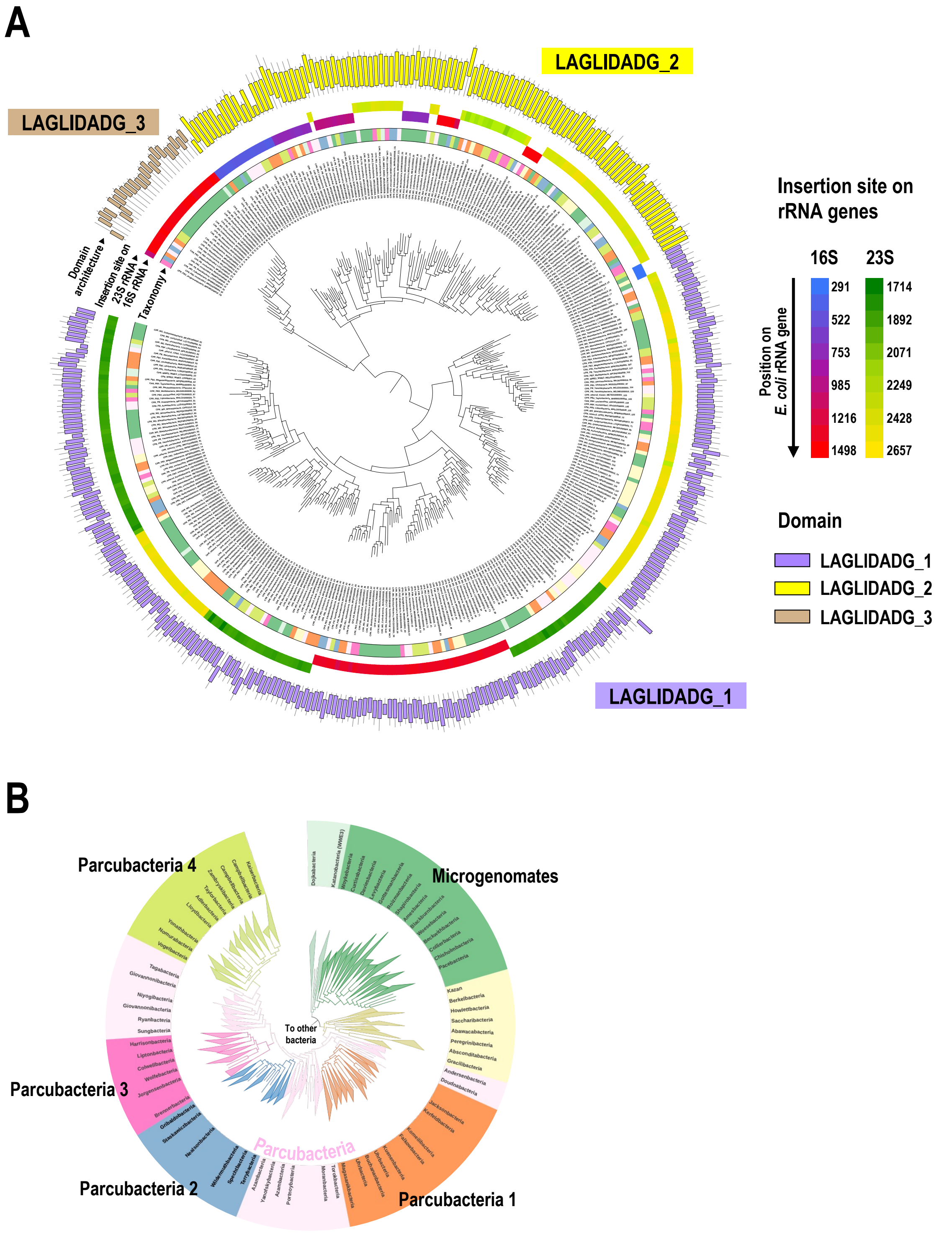

Fig S6. Phylogenetic tree of LAGLIDADG proteins encoded by rRNA genes of CPR bacteria. (A) Phylogenetic tree constructed based on LAGLIDADG proteins (n = 427) encoded by the rRNA genes of CPR bacteria using the maximum likelihood method and rooted using midpoint rooting. The taxonomic classification of CPR phyla is represented in the inner circle, colored according to Fig. S4B. Insertion sites of the protein-encoding ISs in each rRNA gene are indicated by a gradient of color (middle two circles). Schematic representations of the protein domain architectures are shown in the outermost circle. (B) Phylum-level phylogenetic tree of CPR bacteria (1), colored according to taxonomic group.

```
     NdeI
1    ATGAACGTTGTGAATCAGCAGGAACGTCGCATTAGTCGTCGCAAATATCTGTTCTACTAC   60
     M  N  V  V  N  Q  Q  E  R  R  I  S  R  R  K  Y  L  F  Y  Y

61   ATGGCCGGATTTGTCGAAGGTGATGGCTGCTTTAGCGTGTCGATCAAGAAATACAAACAG   120
     M  A  G  F  V  E  G  D  G  C  F  S  V  S  I  K  K  Y  K  Q

121  ATGAAATTCGGTTGGGTCGTTGATCCGATGTTTTCCGTATATCAGCACAAAAGCAACAAA   180
     M  K  F  G  W  V  V  D  P  M  F  S  V  Y  Q  H  K  S  N  K

181  ATCATGTTAGAATTGTTCCAGAAAGAACTCCATTGTGGGTATATTGTTAAGAAGAAAGGG   240
     I  M  L  E  L  F  Q  K  E  L  H  C  G  Y  I  V  K  K  K  G

241  AAAAGCGATGTGCTTGTGTATGTGGTAGACAATCGTCGTACCCTGGAAGAGAAAATCCTG   300
     K  S  D  V  L  V  Y  V  V  D  N  R  R  T  L  E  E  K  I  L

301  CCATTCTTTCGGAAACACGTTTTGCTGGGCAAGAAGTGGAATGACTTTCTGATCTTTGAG   360
     P  F  F  R  K  H  V  L  L  G  K  K  W  N  D  F  L  I  F  E

361  AAAATTGTGCTTATGATGAAAGACAAGAAACATCTCCAACGCCAAGGCCTGAAAGAGATT   420
     K  I  V  L  M  M  K  D  K  K  H  L  Q  R  Q  G  L  K  E  I

421  GTCAAATTAGCGTTCAAAATGAACCAACAGGGTAAAGGCCGCAAATATTCAGAGAAAGAA   480
     V  K  L  A  F  K  M  N  Q  Q  G  K  G  R  K  Y  S  E  K  E
                                                 XhoI
481  ATTCTGGAAGATCTGTCGGAATCTCCGGAAACGATTCGCCTCGAGCACCACCACCACCAC   540
     I  L  E  D  L  S  E  S  P  E  T  I  R  L  E  H  H  H  H  H
                                                     His-Tag
541  CACTGA                                                         546
     H  *
```

Fig S7. Nucleotide sequence of artificial *I-ShaI* gene used in the experiment and its deduced amino acid sequence. The nucleotide sequence of the artificial *I-ShaI* gene, in which codon usage was optimized for its efficient expression in *E. coli* (upper line), and the deduced amino acid sequence (lower line) are shown. The termination codon is shown with a star. Numbers indicate nucleotide positions.

Reference:

1. Jaffe AL, Castelle CJ, Matheus Carnevali PB, Gribaldo S, Banfield JF. 2020. The rise of diversity in metabolic platforms across the Candidate Phyla Radiation. *BMC Biol*. 18(1):69.
